# Additivity of Pyrethroid Actions on Sodium Influx in Cerebrocortical Neurons in Primary Culture

**DOI:** 10.1289/ehp.1003394

**Published:** 2011-06-10

**Authors:** Zhengyu Cao, Timothy J. Shafer, Kevin M. Crofton, Chris Gennings, Thomas F. Murray

**Affiliations:** 1 Creighton University, School of Medicine, Department of Pharmacology, Omaha, Nebraska, USA; 2 Integrated Systems Toxicology Division, National Health and Environmental Effects Research Laboratory, Office of Research Development, U.S. Environmental Protection Agency, Research Triangle Park, North Carolina, USA; 3 Solveritas, LLC, Richmond, Virginia, USA

**Keywords:** additivity, efficacy, potency, pyrethroid, sodium influx

## Abstract

Background: Pyrethroid insecticides bind to voltage-gated sodium channels and modify their gating kinetics, thereby disrupting neuronal function. Although previous work has tested the additivity of pyrethroids *in vivo*, this has not been assessed directly at the primary molecular target using a functional measure.

Objectives: We investigated the potency and efficacy of 11 structurally diverse food-use pyrethroids to evoke sodium (Na^+^) influx in neurons and tested the hypothesis of dose additivity for a mixture of these same 11 compounds.

Methods: We determined pyrethroid-induced increases in Na^+^ influx in primary cultures of cerebrocortical neurons using the Na^+^-sensitive dye sodium-binding benzofuran isophthalate (SBFI). Concentration-dependent responses for 11 pyrethroids were determined, and the response to dilutions of a mixture of all 11 compounds at an equimolar mixing ratio was assessed. Additivity was tested assuming a dose-additive model.

Results: Seven pyrethroids produced concentration-dependent, tetrodotoxin-sensitive Na^+^ influx. The rank order of potency was deltamethrin > *S*-bioallethrin > β-cyfluthrin > λ-cyhalothrin > esfenvalerate > tefluthrin > fenpropathrin. Cypermethrin and bifenthrin produced modest increases in Na^+^ influx, whereas permethrin and resmethrin were inactive. When all 11 pyrethroids were present at an equimolar mixing ratio, their actions on Na^+^ influx were consistent with a dose-additive model.

Conclusions: These data provide *in vitro* relative potency and efficacy measurements for 7 pyrethroid compounds in intact mammalian neurons. Despite differences in individual compound potencies, we found the action of a mixture of all 11 pyrethroids to be additive when we used an appropriate statistical model. These results are consistent with a previous report of the additivity of pyrethroids *in vivo*.

Pyrethroids are synthetic insecticidal compounds that resemble pyrethrins—natural  toxins present in the flowers of *Chrysanthemum* species. Pyrethroids are used worldwide to control insect pests (Casida and Quistad 1998), and the use of pyrethroids has increased by as much as 25% since 2000 (Amweg et al. 2005; Spurlock and Lee 2008; U.S. Environmental Protection Agency 1999). This increased use carries the potential for increased human exposure, including pregnant women, infants, and children, to individual or multiple pyrethroid compounds (Morgan et al. 2007; Whyatt et al. 2002).

Based on their chemical structure and signs of toxicity at high doses (Verschoyle and Aldridge 1972, 1980), pyrethroids are classified into two major groups. Type I pyrethroids lack a cyano group at the α-carbon of the 3-phenoxybenzyl alcohol moiety and produce a poisoning syndrome consisting of hyperexcitation, tremors, and convulsions (T syndrome). Type II pyrethroids have a cyano group at the α-carbon of the 3-phenoxybenzyl alcohol moiety and produce an acute, high-dose syndrome consisting of hypersensitivity, salivation, choreoathetosis, and clonic seizures (CS syndrome) (Soderlund et al. 2002; Wolansky and Harrill 2008). The primary molecular targets underlying pyrethroid-induced toxicity are voltage-gated sodium channels (VGSCs) of insects and vertebrates (Narahashi 1996; Soderlund et al. 2002). Pyrethroids enhance sodium channel activity by shifting activation to more negative membrane potentials and by inhibiting channel inactivation; type II compounds typically produce longer lasting delays in channel inactivation than do type I compounds (Narahashi 1996). VGSCs are responsible for the rapid influx of sodium that underlies the rising phase of the action potential in most electrically excitable cells, including neurons, and the difference in actions of type I and type II pyrethroids on VGSC kinetics is hypothesized to contribute significantly to the T and CS syndromes (Narahashi 1992).

Recently, Wolansky et al. (2009) predicted the effects of a low-dose mixture of pyrethroid compounds on rat behavior (motor activity) using a dose-additive model. An interesting finding in that study was that both type I and type II pyrethroids were dose additive. The use of motor activity as an end point, however, has the disadvantage of being a nondiagnostic end point (i.e., activation of many neural pathways and processes decrease motor activity). Therefore, whether this *in vivo* finding is due to dose additivity at the molecular target (e.g., VGSCs) remains to be determined.

Little information is available regarding dose additivity of pyrethroids at VGSCs. Studies of binary combinations of pyrethroids indicate that type I and type II compounds may act competitively on VGSCs (Motomura and Narahashi 2001; Song et al. 1996), but these studies did not examine concentration–response relationships and were not designed to test the assumption of additivity. Therefore, in the present study we determined the relative potencies of 11 pyrethroids for stimulation of sodium (Na^+^) influx in cerebrocortical neurons and tested the hypothesis that alterations in Na^+^ influx by a mixture of 11 pyrethroids would be dose additive.

## Materials and Methods

*Materials.* The pyrethroids (and purity) used were deltamethrin (98.9%), β-cyfluthrin (99.2%), cypermethrin (88%), permethrin (96%), bifenthrin (95%), esfenvalerate (98.6%), λ-cyhalothrin (87.7%), tefluthrin (92.6%), fenpropathrin (91.8%), resmethrin (92.3%), and *S*-bioallethrin (95.6%). These compounds were from the same lots used previously by Wolansky et al. (2006, 2009). Pyrethroids are chiral compounds and exist in multiple stereoisomers that have different activities on sodium channel function (Soderlund et al. 2002). Of the compounds used in the present study, only deltamethrin, bifenthrin, and tefluthrin were available as a single isomer; all other compounds were mixtures of two or more stereoisomers. Complete information on pyrethroid sources and isomer composition has been described by Wolansky et al. (2006) and is available in Supplemental Material (http://dx.doi.org/10.1289/ehp.1003394). TTX was purchased from Tocris Bioscience (Ellisville, MO) and veratridine was from Sigma-Aldrich (St. Louis, MO).

*Compound preparation.* Each pyrethroid was dissolved in dimethyl sulfoxide (DMSO) at a stock concentration of 100 mM. An aliquot of stock solution of each pyrethroid was then diluted using DMSO to give a series of secondary stock solutions of 30, 10, 3, 1, 0.3, 0.1, and 0.03 mM. These secondary stock solutions were kept at –20°C in the dark. Before each experiment, an equal volume of pluronic acid F-127 (20% in DMSO; to improve the solubility of pyrethroids) was then mixed with the secondary stock solution and diluted 250 times using Locke’s buffer (8.6 mM HEPES, 5.6 mM KCl, 154 mM NaCl, 5.6 mM glucose, 1.0 mM MgCl_2_, 2.3 mM CaCl_2_, 0.0001 mM glycine; pH 7.4). This dilution in Locke’s buffer resulted in solutions of pyrethroid that were 4× the desired final concentrations. To examine the effect of a mixture of pyrethroids on sodium flux, a mixture of all 11 pyrethroids was prepared that contained equimolar concentrations (1 µM) of each compound in Locke’s buffer. The effects of this mixture were then tested at different dilution levels: 100%, 66%, 50%, 33%, 10%, 5%, and 1%; 0.2% DMSO was added to separate wells as the control.

*Cerebrocortical neuron culture.* All animal use protocols were approved by the Creighton University Institutional Animal Care and Use Committee. Animals were treated humanely and with regard for alleviation of suffering. Cerebrocortical neuron cultures were obtained from embryonic day 16 Swiss-Webster mice as described previously (Cao et al. 2008). Cerebrocortical cultures were used in experiments between 8 and 10 days *in vitro*.

*Sodium influx measurement.* Neuronal intracellular Na^+^ concentration ([Na^+^]*_i_*) was determined by sodium-binding benzofuran isophthalate (SBFI) fluorescence measurement as described previously (Cao et al. 2008). The background fluorescence of each well was measured in a FLEXstation II (Molecular Devices, Sunnyvale, CA, USA) before dye loading. Cells were then incubated for 1 hr at 37°C with dye loading buffer (50 μL/well) containing 10 µM SBFI acetoxymethyl ester (AM) and 0.02% Pluronic F-127 and then washed five times with Locke’s buffer, leaving a final volume of 150 μL in each well. Cells were excited at 340 and 380 nm using a FLEXstation II, and Na^+^-bound SBFI emission was detected at 505 nm. Fluorescence readings were taken once every 5 sec for 1 min to establish the baseline; 50 μL of test compound solution (4×) was then added to each well, yielding a final volume of 200 μL. The cells were exposed to test compounds for another 4.5 min.

*Statistical analysis.* For sodium influx data, fluorescence values over time were summed and the baseline subtracted. We determined the actions of tetrodotoxin (TTX) on Na^+^ influx using a one-way analysis of variance (ANOVA), followed by comparisons between specified treatment groups using a Bonferroni comparison. To determine the relative potency of each chemical and test for additivity, we applied the flexible single-chemical required method of analysis (Gennings et al. 2004) to the data, because preliminary analysis indicated that the concentration responses for each chemical did not have a common maximum effect parameter (i.e., they had distinct efficacies). Details of the analysis strategy are provided in the Supplemental Material (http://dx.doi.org/10.1289/ehp.1003394). This approach has been demonstrated to be suitable for examining additivity of complex mixtures, including those that may contain one or more components that are inactive or have little activity (Crofton et al. 2005; Moser et al. 2005, 2006). The full model (a nonlinear logistic model parameterized as given in Supplemental Material, Equation 2) was used to simultaneously fit all of the single chemical data as well as the fixed-ratio mixture data. For parsimony, using a backward elimination criterion, the chemicals were grouped based on their maximum effect parameter (i.e., chemicals with similar maximum effect parameter estimates were grouped to have a common parameter) thereby reducing the number of parameters. The output of the full model was used to calculate EC_30_ (30% effective concentration) values and 95% confidence intervals (CIs) for each individual pyrethroid that had a significant concentration–response relationship. (The EC_30_ was defined as the concentration of pyrethroid that induced 30% of the maximal Na^+^ influx based on the estimated range of the data.) We selected the EC_30_ because this parameter has been used previously to determine relative potencies for pyrethroids (Wolansky et al. 2006, 2009). Thus, our metric for relative potency comparisons are the same as those used previously. Based on these EC_30_ values, the relative potencies of the individual chemicals were determined using deltamethrin as an index chemical. We selected deltamethrin as an index chemical because *a*) there is an extensive *in vitro* and *in vivo* database for this compound, *b*) it was the most potent compound in this assay, and *c*) it previously has been used as an index chemical in other comparisons of relative potency (Wolansky et al. 2006) and mixture effects (Wolansky et al. 2009). A reduced model was estimated under the hypothesis of additivity using all of the data, but only single-chemical model parameters were used when the prediction under additivity was used for the mixture data (see Supplemental Material, Equation 3). That is, the reduced model simultaneously estimated concentration–response curves for the single chemicals, and used these to predict the mixture concentration–response curve using dose addition. We compared the actual mixture data with the predicted mixture concentration–response curve, and we used a likelihood ratio test to test the hypothesis of additivity using an *F*-distribution.

## Results

**Figure 1:**
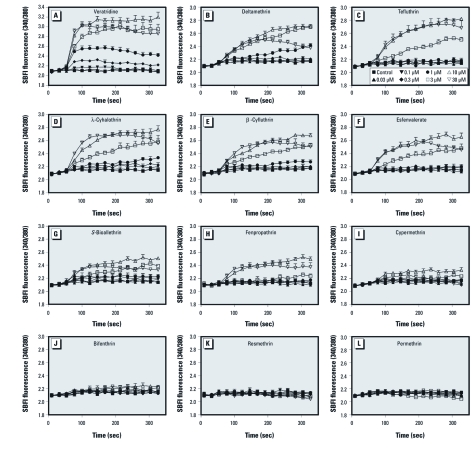
Dose–response and time–response relationships for Na^+^ influx ([Na^+^]*_i_*) induced after exposure to 0.2% DMSO or different concentrations of veratridine (*A*), deltamethrin (*B*), tefluthrin (*C*), λ-cyhalothrin (*D*), β-cyfluthrin (*E*), esfenvalerate (*F*), *S*-bioallethrin (*G*), fenpropathrin (*H*), cypermethrin (*I*), bifenthrin (*J*), resmethrin (*K*), and permethrin (*L*). Each data point represents the mean ± SE from at least two different cultures performed in duplicate (*n* = 4–7/treatment).

*Pyrethroids produce sodium influx in cerebrocortical neurons.* A comprehensive study of the relative potencies of pyrethroids on VGSCs in intact mammalian neurons has not previously been undertaken. We therefore compared the ability of 11 structurally diverse food-use pyrethroids to evoke sodium influx in cerebrocortical neurons using SBFI, a sodium-sensitive fluorescent dye. Seven of the 11 pyrethroids tested (tefluthrin, deltamethrin, λ-cyhalothrin, β-cyfluthrin, esfenvalerate, *S*-bioallethrin, and fenpropathrin) produced a rapid and concentration-dependent elevation in neuronal Na^+^ influx (Figure 1). The maximum responses for Na^+^ influx produced by pyrethroids were all lower than that of 10 µM veratridine (Figure 1A), a plant-derived sodium channel neurotoxin site 2 agonist. Two of the 11 pyrethroids tested, cypermethrin and bifenthrin, caused only modest increases in Na^+^ flux, and two others, resmethrin and permethrin, did not stimulate Na^+^ influx.

**Figure 2:**
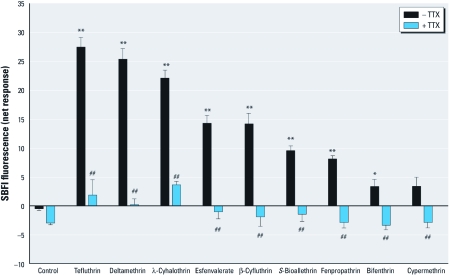
TTX blocked pyrethroid-induced elevation of Na^+^ influx in neocortical cultures. Cultures were loaded with SBFI and washed, and baseline fluorescence was determined for 100 sec. Individual wells were then treated with vehicle (0.2% DMSO), vehicle + 1 µM TTX, 10 µM of the indicated pyrethroids, or 10 µM of the pyrethroids + 1 µM TTX. Data (mean ± SE; *n* = 3–6/treatment) were obtained from two different cultures performed in duplicate. In the absence of pyrethroid (control), the negative SBFI fluorescence in the presence of TTX indicates an effect of TTX on basal activity of VGSCs. In all cases, TTX significantly reduced Na^+^ flux stimulated by pyrethroids. Two-way ANOVA indicated a significant main effect of pyrethroid, a significant main effect of TTX treatment, and a significant interaction. Step-down ANOVAs followed by Bonferroni’s comparison indicated a significant effect of all pyrethroids versus control except for cypermethrin and a significant effect of TTX on all treatments (control and pyrethroid compounds) except control. *p < 0.05 and **p < 0.01 for pyrethroid compared with control. ^##^*p* < 0.01 for pyrethroid + TTX compared with pyrethroid alone.

To confirm the role of VGSCs in pyrethroid-induced increases of Na^+^ influx, we examined the influence of TTX (1 µM) on the responses produced by the nine active pyrethroids (10 µM). All compounds except cypermethrin produced statistically significant increases in Na^+^ influx (Figure 2). TTX eliminated this pyrethroid-induced Na^+^ influx, including the modest Na^+^ influx produced by bifenthrin and cypermethrin. These results demonstrate that the observed elevation of neuronal [Na^+^]*_i_* induced by these nine pyrethroids depended on the activation of TTX-sensitive VGSCs.

**Figure 3:**
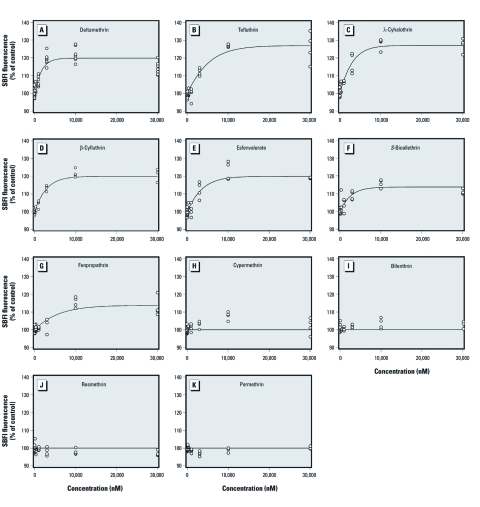
Concentration–response relationships for 11 pyrethroids-induced Na^+^ influx: (*A*) deltamethrin, (*B*) tefluthrin, (*C*) λ-cyhalothrin, (*D*) β-cyfluthrin, (*E*) esfenvalerate, (*F*) *S*-bioallethrin, (*G*) fenpropathrin, (*H*) cypermethrin, (*I*) bifenthrin, (*J*) resmethrin, and (*K*) permethrin. Primary cultures of cerebrocortical neurons were loaded with SBFI and then treated with 0.2% DMSO or the indicated concentrations of each pyrethroid for 4.5 min. The levels of fluorescence intensity were summed across the 4.5‑min period, and the background fluorescence was subtracted. Data were then fitted to a nonlinear logistic model to estimate the potency. Data are presented as a percentage of control values, with controls set at 100%. Data points represent individual determinations (wells).

The concentration–response relationships for all 11 pyrethroids are illustrated in Figure 3, and Table 1 provides the model parameters for the 11 individual compounds. Fenpropathrin and *S*-bioallethrin had the smallest estimated maximum effect parameter (113.7; i.e., roughly a 14% increase above control; see α_6,10_ parameters in Table 1), followed by β-cyfluthrin, deltamethrin, and esfenvalerate (with maximum effects approximately 20% above control; see α_1,4,5_); λ-cyhalothrin, tefluthrin, and the mixture produced the highest maximum increases, estimated to be about 27% above control (α_7,11,mix_). These data indicate that pyrethroids have distinct efficacies. Four compounds— cypermethrin, bifenthrin, permethrin, and resmethrin—did not produce significant concentration–response profiles. The small, TTX-sensitive increases in Na^+^ influx caused by cypermethrin and bifenthrin were not sufficient to model a concentration–response relationship to quantify their potency. The rank order of potency for the remaining seven compounds was deltamethrin > *S*-bioallethrin > β-cyfluthrin > λ-cyhalothrin > esfenvalerate > tefluthrin > fenpropathrin (Table 2).

**Table 1:** Estimated model parameters from the full model for both the single-chemical and mixture data fitted simultaneously with mean squared error = 0.11.

Chemical abbreviation	Full model							Additivity model		
	Chemical	Parameter	Estimate	SE	*p*-Value	Estimate	SE
				α_1,4,5_		119.9		0.71		< 0.001		119.9		0.79
				α_6,10_		113.7		1.06		< 0.001		113.7		1.16
				α_7,11,mix_		126.9		0.95		< 0.001		127.7		1.27
β-Cyfluthrin		BCF		β_1_ (BCF)		0.40		0.08		< 0.001		0.40		0.08
Bifenthrin		BIF		β_2_ (BIF)		0						0		
Cypermethrin		CPM		β_3_ (CPM)		0						0		
Deltamethrin		DEL		β_4_ (DEL)		0.65		0.10		< 0.001		0.65		0.10
Esfenvalerate		ESF		β_5_ (ESF)		0.32		0.06		< 0.001		0.32		0.06
Fenpropathrin		FEN		β_6_ (FEN)		0.19		0.06		0.001		0.19		0.05
λ-Cyhalothrin		LCH		β_7_ (LCH)		0.39		0.06		< 0.001		0.38		0.07
Permethrin		PER		β_8_ (PER)		0						0		
Resmethrin		RES		β_9_ (RES)		0						0		
*S*-Bioallethrin		S-BIO		β_10_ (S-BIO)		0.43		0.13		< 0.001		0.46		0.14
Tefluthrin		TEF		β_11_ (TEF)		0.23		0.04		< 0.001		0.22		0.03
Mixture		Mixture		θ_mix_		0.28		0.03		< 0.001				
The full model is given in Supplemental Material, Equation 2 (http://dx.doi.org/10.1289/ehp.1003394). There is no significant departure from additivity using a likelihood ratio test [*F* = 0.55; df = (1, 428); *p* = 0.459].														

**Table 2:** Estimated EC_30_ values (with large sample 95% CIs) and relative potencies for each of the 11 pyrethroids.

Chemical	EC_30_ estimate (nM)	95% CI	Relative potency*a*
β-Cyfluthrin	1,046		645–1,447		0.61
Bifenthrin					
Cypermethrin					
Deltamethrin	635		438–832		1.00
Esfenvalerate	1,301		793–1,809		0.49
Fenpropathrin	2,047		819–3,274		0.31
λ-Cyhalothrin	1,101		765–1,437		0.58
Permethrin					
Resmethrin					
*S*-Bioallethrin	932		389–1,475		0.68
Tefluthrin	1,891		1,317–2,464		0.34
Mixture	1,568		1,279–1,857		0.41
**a**Relative potencies for the effects of 11 pyrethroids on Na^+^ influx based on deltamethrin (EC_30_ = 635 nM) as the index chemical. Relative potency was calculated as the ratio of the EC_30_ for deltamethrin over the EC_30_ for each chemical. The EC_30_ values and 95% CIs were determined from the four to seven measurements presented in Figure 1.					

**Figure 4:**
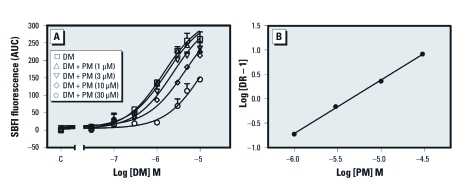
(*A*) Concentration–response relationships for deltamethrin (DM)-induced elevation of neuronal Na^+^ influx in the presence of vehicle control (C) or different concentrations (1, 3, 10, and 30 µM) of permethrin (PM). (*B*) Schild analysis for PM as an antagonist of the pyrethroid binding site on VGSC α-subunits. Abbreviations: AUC, area under the curve; DR, dose ratio. Experiments were repeated three times with similar results.

*Permethrin acts as a low-efficacy partial agonist in the presence of deltamethrin.* Permethrin and resmethrin were without detectable actions on Na^+^ influx; thus, we sought to confirm that they interacted with VGSCs in mouse neurons. Jacques et al. (1980) demonstrated that although *cis*- permethrin and bioresmethrin did not potentiate veratridine-induced ^22^Na^+^ uptake in neuroblastoma cells, they did produce a concentration-dependent reduction of ^22^Na^+^ uptake induced by a combination of veratridine and kadethrin. We therefore tested the influence of different concentrations of permethrin on deltamethin-induced sodium influx to confirm that permethrin interacts with VGSCs in our preparation. Permethrin produced a concentration-dependent rightward shift of deltamethrin concentration–response curves for sodium influx (Figure 4A). Accordingly, we analyzed these data by Schild regression. The slope value from the linear regression was 1.10 (95% CI, 0.99–1.21), indicating that permethrin, although lacking efficacy, interacts competitively with the same site on the sodium channel α-subunit as deltamethrin. The affinity of permethrin derived from the Schild pA_2_ value was 4.43 µM (95% CI, 3.88–5.04) (Figure 4B). We therefore included permethrin, bifenthrin, cypermethrin, and resmethrin in the mixture study, even though they did not produce significant concentration–response relationships.

**Figure 5:**
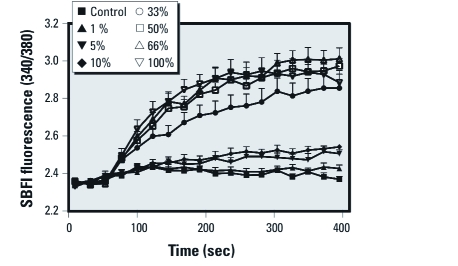
Actions of a mixture of 11 pyrethroids on SBFI fluorescence (340/380) in mouse cortical neurons. An equally weighted mixture of all 11 pyrethroids was prepared such that the concentration of each pyrethroid in the mixture was 1 µM. After collection of baseline data, mouse cerebrocortical neurons were exposed to 0.2% DMSO or 1%, 5%, 10%, 33%, 50%, 66%, and 100% dilutions of this mixture, and SBFI fluorescence was monitored for an additional 4.5 min. Values shown are mean ± SE (*n* = 9 wells from two separate plates per treatment).

**Figure 6:**
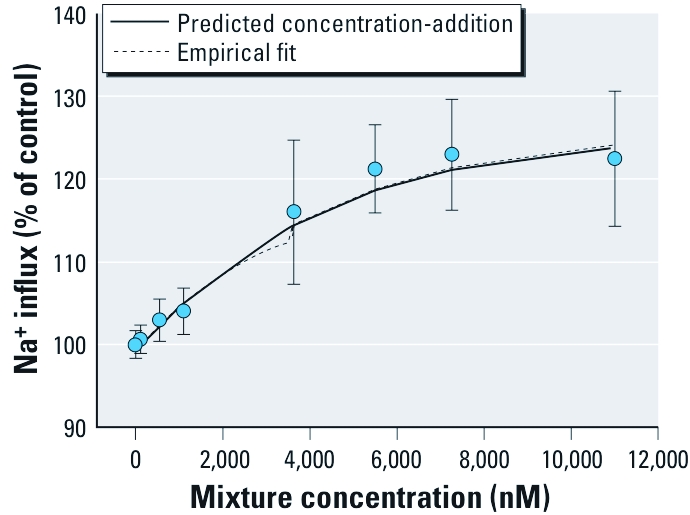
Observed sample means (circles) and model-predicted mean Na^+^ influx for a mixture of 11 pyrethroids. Means for the mixture were determined by summing the levels of fluorescence intensity across the 4.5‑min period and subtracting the background fluorescence. Mixture data were then fitted to a nonlinear logistic model, and are presented as the mean ± SD percentage of control values, with controls set at 100%. The predicted response for the mixture, using only single chemical parameters, is not significantly different from the full model, as indicated by the empirical fit. Using this approach, the mean under additivity is estimated for mean values less than the minimum–maximum effect parameter; conservatively, mean values for concentrations above this constraint were set to the full model mean estimates.

*Effects of the pyrethroid mixture on Na^+^ influx in cerebrocortical neurons.* A mixture of all 11 pyrethroids produced an increase in intracellular Na^+^ that was both rapid and efficacious (Figure 5). Using the flexible single-chemical required method of testing additivity, data are consistent with dose additivity (Figure 6; hypothesis of additivity is not rejected; *p* = 0.459). The similarity of the parameter estimates for the full model compared with the reduced model (Table 1) demonstrate the predictability of the mean responses for the mixture data using dose addition and single-chemical information.

## Discussion

Experiments in the present study were designed to test the hypothesis that the actions of a pyrethroid mixture on Na^+^ influx in mammalian neurons could be predicted by a dose-additive model. To test this hypothesis, we first needed to determine the concentration–response relationship for each of the individual pyrethroids in the mixture. Using the same 11 pyrethroids that have previously been tested individually (Wolansky et al. 2006) and in mixtures (Wolansky et al. 2009) *in vivo*, we demonstrated that they stimulate TTX-sensitive Na^+^ influx in primary cultures of cerebrocortical neurons with differing potencies and efficacies. Of the 11 compounds examined, 7 caused concentration-dependent increases in Na^+^ influx, whereas 2 compounds (cypermethrin and bifenthrin) caused small increases, and 2 (permethrin and resmethrin) were without effect. When the action of an equimolar mixture of these 11 pyrethroids on Na^+^ influx was assessed, the effect of the mixture was predicted by a dose-additive model. This is consistent with the results of an *in vivo* mixture study using motor activity as an end point (Wolansky et al. 2009) and indicates that both the behavioral and pharmacological effects of pyrethroid mixtures are dose additive.

VGSCs are well-established targets of pyrethroid effects and clearly contribute to the insecticidal and toxicological actions of these compounds (Narahashi 1996). Thus, effects of individual pyrethroids and pyrethroid mixtures on VGSCs reflect the actions of these compounds at a key molecular target relevant to their toxicity. The use of a sodium-sensitive fluorescent dye allowed us to determine the effects of 11 pyrethroids in a high-throughput manner without disruption of cellular integrity, as described previously (Cao et al. 2008). This pyrethroid-induced Na^+^ influx depended on the activation of TTX-sensitive VGSCs inasmuch as the response was abrogated by TTX, a sodium channel blocker. The ability of SBFI measurements to detect deltamethrin effects in the nanomolar range indicates that this assay is similar in sensitivity to membrane potential (Eells et al. 1993), ^22^Na^+^ flux (Ghiasuddin and Soderlund 1985), and electrophysiological (Meyer et al. 2008; Shafer et al. 2008) measures, all of which also detect deltamethrin effects in the nanomolar range.

The relative potencies observed in the present *in vitro* study are not identical to those reported by Wolansky et al. (2006). In that *in vivo* study, type II compounds were generally more potent than type I compounds in both Na^+^ influx and motor activity assays. However, the range of relative potencies was much larger *in vivo* [230-fold (Wolansky et al. 2006)] than *in vitro* [3.3-fold present study)], and the rank order of relative potencies *in vitro* differs from that observed *in vivo*. Several factors are likely to account for these differences. The smaller range of relative potencies *in vitro* reflects the fact that potencies could not be determined for four of the compounds tested. These included three (permethrin, resmethrin, and cypermethrin) of the least potent compounds *in vivo* (Wolansky et al. 2006); thus, the range of relative potencies *in vitro* may have been greater if potency estimates for these compounds could have been made. The *in vitro* system also differs from the *in vivo* system in two other important aspects: it lacks significant metabolic capability, and it consists of a single tissue. The relative potencies from *in vivo* studies are influenced by several factors not present *in vitro*, including metabolism, contributions of multiple neuronal pathways, actions on a greater number of VGSC isoforms than may be present *in vitro*, and potentially greater contributions of secondary effects of pyrethroids (e.g., neurotransmitter release). Comprehensive data sets from other *in vitro* studies permitting determination of relative potencies of pyrethroids are generally lacking. We recently demonstrated that this same set of 11 pyrethroids induced Ca^2+^ influx in mouse primary cultures of cerebrocortical neurons with distinct potencies (Cao et al. 2011) that are well correlated with those reported here for Na^+^ influx. This is to be expected inasmuch as Ca^2+^ influx is completely dependent on activation of VGSCs by pyrethroids (Cao et al. 2011). Pyrethroids also produced distinct maximum responses in the increase of neuronal Na^+^ influx in cerebrocortical neurons. The different efficacies of pyrethroids on Na^+^ influx are consistent with previous reports that pyrethroids display distinct efficacies to either augment [^3^H]BTX (batrachotoxin)-specific binding (Brown et al. 1988; Trainer et al. 1993) or to elevate brain-derived neurotropic factor (*BDNF*) mRNA expression levels (Imamura et al. 2006).

Four compounds (cypermethrin, bifenthrin, permethrin, and resmethrin) had little or no effect on Na^+^ influx in the present study. Cypermethrin and bifenthrin caused small TTX-sensitive responses, indicating that they do interact with VGSCs in cortical neurons, but did not produce a response of sufficient magnitude to be fitted by a logistic concentration–response function. These two compounds also elicited TTX-sensitive Ca^2+^ influx in cerebrocortical neurons (Cao et al. 2011), which further indicates that they activate VGSCs. Our demonstration of the ability of permethrin to antagonize  deltamethrin-induced VGSC activation indicates that permethrin also interacts with VGSCs in cerebrocortical neurons. These results agree with reports of an interaction between type I and type II pyrethroids on both whole-cell (Song et al. 1996) and single-channel (Motomura and Narahashi 2001) sodium currents. Similarly, permethrin was unable to either augment [^3^H]BTX-specific binding (Brown et al. 1988; Trainer et al. 1993) or stimulate *BDNF* mRNA expression in neurons (Imamura et al. 2006). These data are consistent with results in neuroblastoma cells showing that *cis*-permethrin and bioresmethrin inhibited ^22^Na^+^ influx induced by a combination of veratridine and kadethrin (Jacques et al. 1980), and with a report by Brown et al. (1988) demonstrating that permethrin produced a concentration-dependent reversal of deltamethrin-induced stimulation of [^3^H]BTX-specific binding to VGSCs. Thus, in a variety of *in vitro* preparations, permethrin and resmethrin are inactive alone but clearly interact with VGSCs. These data parallel an earlier report (Cao et al. 2008) demonstrating partial agonism at neurotoxin site 5 in that treatment of neurons with a combination of a high-efficacy activator (brevetoxin 1) and a low-efficacy activator (gambierol) resulted in an inhibition of the response to the high-efficacy compound by the low-efficacy compound. This profile for permethrin is therefore typical of a low-efficacy partial agonist that can behave as an inhibitor in the presence of a high- efficacy agonist (Cao et al. 2008). Moreover, the inference that permethrin behaves as a low-efficacy agonist is consistent with studies that demonstrate that permethrin increases open probability of VGSCs.

Although previous studies have indicated that interactions between pyrethroids occur *in vitro*, they have not rigorously tested the hypothesis of additivity or examined interactions between multiple pyrethroids. The present study was designed specifically to test the hypothesis of dose additivity for the effects of multiple pyrethroids. Each compound was present in the mixture at an equimolar concentration (~ 1 µM at the highest mixture dilution), which was ≤ EC_30_ values for six of the seven individual compounds. Thus, the concentrations used in the mixture were relatively low on the concentration–effect curves for each individual compound. In addition, the mixture contained four compounds that were without significant concentration– response effects on Na^+^ influx. The mixture caused concentration-dependent increases in Na^+^ influx, with a maximal effect that was equivalent to the most efficacious of the individual compounds. Thus, the hypothesis that pyrethroid actions on Na^+^ influx were dose additive was not rejected. The effects of mixtures of pyrethroids are therefore additive at the behavioral (Wolansky et al. 2009) as well as the cellular level. The statistical model used in the present study is well suited for predicting the additivity of mixtures that contain components that vary widely in their activity, from very active to completely inactive (Crofton et al. 2005), such as shown here with the pyrethroids. In this case, the four compounds present were of such low activity that potency values could not be estimated. One (permethrin) inhibited deltamethrin actions when these two compounds were present in a binary mixture. In the 11-chemical mixture, the net effect of all the compounds in the mixture did not differ from additivity; this result reflects the combined action of all 11 compounds but does not make any predictions or conclusions regarding the interactions of individual pairs of compounds in the mixture. If the objective of the present study were to identify and characterize the interaction profile of all chemicals, the study design and size would be much larger: A factorial design with only three levels of each compound would require 3^11^ = 177,147 dose groups. With only two dose groups per chemical, 2,048 dose groups would be required for a full factorial design. The fixed-ratio ray design used here is a more focused and efficient design to test for departure from additivity at relevant mixtures of interest. Its limitation is that subsets of the 11 chemicals may interact, but the overall impact agrees with additivity. The design and analysis strategy herein cannot detect or characterize this scenario.

Our results differ from previous reports where actions of pyrethroids on single-channel  (Song and Narahashi 1996) or whole-cell (Song et al. 1996) sodium currents as well as large-conductance chloride channels (Burr and Ray 2004) appeared to be antagonistic between type I and type II compounds. They also differ from one report that deltamethrin and *S*-bioallethrin, at doses that exceeded the LD_50_ (dose lethal to 50%) value, did not produce effect-additive or antagonistic changes in blood pressure, electromyograms, or hippocampal electrophysiological responses (Ray et al. 2006). However, these studies (Burr and Ray 2004; Ray et al. 2006; Song and Narahashi 1996; Song et al. 1996) did not rigorously test the hypothesis of additivity because of limitations in study design and/or  the statistical approach. Dose or effect additivity, synergism, or antagonism between mixtures of chemicals can be reliably determined only by hypothesis testing using rigorous statistical models (Feron and Groten 2002; Gennings et al. 2004; Hertzberg and Teuschler 2002; LeBlanc and Olmstead 2004; Teuschler 2007).

Pyrethroid compounds are chiral and their stereoisomers have differing activities toward VGSCs (Lund and Narahashi 1982). Of the compounds used in the present study, only three (deltamethrin, bifenthrin, and tefulthrin) were single stereoisomers [see Supplemental Material, Table 1 (http://dx.doi.org/10.1289/ehp.1003394)]. We tested each compound individually as a single stereoisomer or group of stereoisomers and then tested the same single stereoisomer or group of stereoisomers in the 11-pyrethroid mixture. The statistical model used data from the individual compounds (whether a single stereoisomer or group of stereoisomers) to predict the dose-additive response and then determined whether the mixture effects significantly differed from this predicted response. In the present study, the results did not differ significantly from additivity, irrespective of the mixture of stereoisomers present.

## Conclusions

The present study demonstrates that pyrethroids stimulate Na^+^ influx in mammalian neurons with distinct potencies and efficacies. Moreover, we demonstrate that alterations in Na^+^ influx by a mixture of food-use pyrethroids can be predicted using a dose-additive model. This is consistent with the actions of these compounds on VGSCs and is in accordance with *in vivo* studies of pyrethroid mixtures.

## Supplemental Material

(72 KB) PDF
