# Supplementary material for: Additivity of Pyrethroid Actions on Sodium Influx in Cerebrocortical Neurons in Primary Culture
Source: Environ Health Perspect. 2011 Jun 10;119(9):1239–46. doi: 10.1289/ehp.1003394 (PMC3230403; doi:10.1289/ehp.1003394)
Supplement: (72 KB) PDF [file ehp.1003394.s001.pdf]

Supplement Material

Additivity of Pyrethroid Actions on Sodium Influx in Cerebrocortical Neurons in Primary Culture

Zhengyu Cao, Timothy J. Shafer, Kevin M. Crofton, Chris Gennings and Thomas F. Murray

Common name	Chemical formula	Isomer composition*	Type	Purity	MW
Deltamethrin	(S)-cyano-(3-phenoxyphenyl)methyl(1R)-cis-3-(2,2-dibromovinyl)-2,2-dimethylcyclopropanecarboxylate	100% (1R3R alphaS)	II	98.9	505.2
Cypermethrin	(R,S)-cyano-(3-phenoxyphenyl)methyl (1R,S)-cis-trans-3-(2,2-dichlorovinyl)-2,2-dimethylcyclopropanecarboxylate mixture of all 8 isomers	48.7% cis, 51.3% trans	II	88..0	416.3
• -Cyfluthrin	(R,S)-cyano-(4-fluoro-3-phenoxyphenyl) methyl-(1RS)-cis,trans-3-(2,2-ichlorovinyl)-2,2-dimethylcyclopropanecarboxylate	2% (1R,3R,aRþ1S,3S,aS) 30–40% (1R,3R,aSþ1S,3S,aR) 3% (1R,3S,aRþ1S,3R,aS) 57–67% (1R,3S,aSþ1S,3R,aR)	II	99.2	434.3
Esfenvalerate	(S)-cyano-(3-phenoxyphenyl)methyl (1S)-2-(4-chlorophenyl)-3-methylbutanoate	85.5% SS isomer 12.0% SR, RR, RS 2.5% other inerts	II	98.6	419.9
• -Cyhalothrin	(R,S)-cyano-(3-phenoxyphenyl) methyl-(Z)-(1R,S)-cis-3-(2-chloro-3,3,3-trifluoro-prop-1-enyl)-2,2-dimethylcyclopropanecarboxylate	50% (S-a-cyano, Z-1R-cis) 50% (R-a-cyano-Z-1S-cis	II	87.7	449.9
Fenpropathrin	(R,S)-cyano-(3-phenoxyphenyl) methyl 2,2,3,3-tetramethylcyclopropanecarboxylate	50% R-cyano 50% S-cyano	I/II	91.8	349.4
Resmethrin	(5-benzyl-3-furyl)methyl (1RS)-cis-trans-2,2-dimethyl-3-(2-methyl prop-1-enyl)-cyclopropanecarboxylate	1:1 ratio of 1R, 1S	I	92.3	338.4
S-Bioallethrin	(S)-3-allyl-2-methyl-4-oxocyclopent-2-enyl (1R)-trans-2,2-dimethyl-3-(2-methylprop-1-enyl)-cyclopropanecarboxylate	5.6% (d-trans-l) 1.7% (l-trans-d,l) <1% (d,l-cis-d,l)	I	95.6	302.4
Permethrin	3-phenoxybenzyl (1R,S)-cis-trans-3-(2,2-dichlorovinyl)-2,2-dimethylcyclopropanecarboxylate	40%cis, 60%trans	I	92.0	391.3
Bifenthrin	2-methylbiphenyl-3-ylmethyl (Z)-(1R)-cis-3-(2-chloro-3,3,3-trifluoroprop-1-enyl)-2,2-dimethylcypropanecarboxylate	100% (Z, 1R cis)	I	89.0	422.9
Tefluthrin	2,3,5,6-tetrafluoro-4-methylbenzyl (Z)-(1R)-cis-3-(2-chloro-3,3,3- trifluoroprop-1-enyl)-2,2-dimethylcypropanecarboxylate	100% (Z, 1R cis)	I	92.6	418.7

Supplemental Material, Table 1;

Chemical information for the pyrethroids used in this study.

Flexible Single Chemical Required method of analysis

A summary of the additive model proposed by Gennings et al (2004) is presented here. Additivity (i.e., zero interaction) as defined by Berenbaum (1985) is related to the isobologram for a combination (mixture) of chemicals (e.g., Loewe and Muischnek, 1926; Loewe, 1953) through the interaction index. To define the interaction index for a combination of *c* chemicals, let E_i represent the concentration of the ith component alone that yields a fixed response, y_0, and let x_i represent the concentration of the ith component in combination with the *c* agents that yields the same response. According to this definition of additivity if the substances combine with zero interaction, then

$$\sum_{i=1}^{c} \frac{x_i}{E_i} = 1. \qquad (1)$$

, then a *Synergism* can be claimed for the mixture of interest if the left-hand side of (1), termed the *interaction index*, is less than 1. *Antagonism* can be claimed for the mxiture if the left-hand side of (1) is greater than 1. This definition of additivity is a general form for dose-addition. In contrast to the toxic equivalency factor (TEF) approach, which assumes common concentration-response slopes across the chemicals under study, the general concentration-addition definition of (1) does not require such an assumption and can be applied to mixtures of chemicals with differing concentration-response relationships.

FSCR: defining additivity using the interaction index set to one

(i) test of additivity:

Different range parameters for each chemical and fixed-ratio mixture can be accommodated in the present approach by using a general form (Gennings et al, 2004) of the additivity model. For convenience in notation, define a model for each of *c* single chemicals and for the fixed-ratio mixture ray with subscript *i*, *i*=1,…, *R* (here, *R*=c+1=12). All single chemical and mixture data were used fit to a nonlinear logistic model , i.e.,

$$\mu_i = \frac{\alpha_i}{1+\exp(-(\beta_0+\beta_i x_i))}, \; i=1,\ldots,R \qquad (2)$$

where $\beta_0 = \log(100/(\alpha_i - 100))$ which constrains the mean to be 100 for the control groups; *x* is the concentration of the ith chemical (*i*=1,…,11) or the total concentration for the mixture ray (*i*=12) divided by 1000 to stabilize the corresponding covariance matrix; α_i is an unknown parameter associated with the maximum effect for the ith single chemical or mixture; and β_i is an unknown parameter associated with the slope for the ith single chemical or mixture. A common intercept parameter was assumed to fix the mean response at the control group to be 100 due to the calculation of the percent control response. For parsimony, a backward elimination criterion was used to combine chemicals into groups with common maximum effect parameters.

The model in (2) was fit simultaneously to all of the single chemical data and the fixed-ratio mixture data. Following Gennings et al, (2004), in order to test for interaction along the fixed-ratio ray of interest using a likelihood-ratio test a reduced additivity model was estimated for comparison. This was accomplished by using only the parameters necessary to estimate the single chemical data from (2) with a constraint of additivity as given in (1) to determine the predicted values along the mixture ray. As

such, $EC_i(\mu)$ is defined as the concentration for the ith chemical alone that produced response μ. From the model in (2),

$$EC_i(\mu) = \frac{\log(\frac{\mu}{\alpha_i - \mu}) - \beta_0}{\beta_i}, \; i=1,\ldots,c.$$

From (1), under additivity, for the mixture of chemicals along the fixed-ratio ray ray with mixing proportion a_i for the ith chemical (i=1,…,c; such that Σa_i=1):,

$$1 = \sum_{i=1}^{c} \frac{a_i t}{EC_i(\mu)} = t \sum_{i=1}^{c} \frac{a_i}{\frac{\log(\frac{\mu}{\alpha_{i(add)} - \mu}) - \beta_{0(add)}}{\beta_{i(add)}}}.$$

Thus, for a specified value of the mean $\mu_{0(add)}$, the corresponding total concentration that yields that mean response under additivity is given by

$$t_{add}(\mu_{0(add)}) = \left[\sum_{i=1}^{c} \frac{a_i}{\frac{\log(\frac{\mu_{0(add)}}{\alpha_{i(add)} - \mu_{0(add)}}) - \beta_{0(add)}}{\beta_{i(add)}}} \right]^{-1}. \qquad (3)$$

Assumpting that variance is proportional to the mean, a quasi-likelihood estimation criterion was used for estimation along the R rays (=c single chemical rays + r mixture rays) using only single chemical model parameters and the constraint in (3), i.e.,

$$Q(add;\tau) = \frac{1}{\tau} \sum_{i=1}^{R} \sum_{j} \sum_{k} (y_{ijk} \log(\mu_{ij(add)}) - \mu_{ij(add)})$$

subject to the constraint given in (3) where y_{ijk} is the kth response from the jth concentration group on the ith ray. The estimation of the additivity model is accomplished by imbedding a grid search into a Newton-Raphson Ridge optimization algorithm. Given candidate values for the model parameters ($\alpha_{i(add)}$, $\beta_{0(add)}$, $\beta_{i(add)}$, i=1,…,c), the grid search is used to find the value of $\mu_{(add)}$ that is associated with each mixture data point where the observed total concentration values, t_{obs}, were such that

$$\left|t_{obs} - t_{add}\right| < \varepsilon \text{ , a small positive value.}$$

Then the Newton-Raphson Ridge algorithm is used to find the constrained quasi-likelihood estimates for the model parameters by maximizing Q*(add)*. A likelihood ratio test of additivity along the fixed-ratio mixture ray(s) of interest is constructed as

$$LLR = \frac{-2(Q(add) - Q(full))/(df_{full} - df_{add})}{\hat{\tau}}$$

which for large samples follows an $F(df_{full}-df_{add}, df_{full})$.

References

Berenbaum MC. 1985. Consequences of synergy between environmental carcinogens. Environ Res 38:310-318.

Gennings C, Carter WH, Jr., Carney EW, Charles GD, Gollapudi BB, Carchman RA. 2004. A novel flexible approach for evaluating fixed ratio mixtures of full and partial agonists. Toxicol Sci 80:134-150.

Loewe S. 1953. The problem of synergism and antagonism of combined drugs. Arzneimittelforschung 3: 285-290.

Loewe S and Muischnek H. 1926. Effect of combinations: mathematical basis of the problem" Arch Exp Pathol Pharmakol 114: 313-326
